# RET (C620R) Mutation in a Hirschsprung Disease Family: A Case Report Unveiling Asymptomatic Pheochromocytoma and Unmanifested Medullary Thyroid Carcinoma

**DOI:** 10.7759/cureus.85803

**Published:** 2025-06-11

**Authors:** Yuko Tanaka, Hiroshi Suzumura, Kan Suzuki, Kazuyuki Ishida

**Affiliations:** 1 Department of Oncology, Dokkyo Medical University Hospital, Tochigi, JPN; 2 Department of Pediatrics, Dokkyo Medical University, Tochigi, JPN; 3 Department of Pediatric Surgery, Dokkyo Medical University, Tochigi, JPN; 4 Department of Diagnostic Pathology, Dokkyo Medical University, Tochigi, JPN

**Keywords:** hirschsprung disease (hd), medullary thyroid carcinoma, multiple endocrine neoplasia type 2a, pheochromocytoma, ret gene variant

## Abstract

*RET *gene variants have been reported in a proportion of patients with familial Hirschsprung disease (F-HSCR), and certain variants are also associated with hereditary medullary thyroid carcinoma (MTC). Clinical guidelines have been developed to support decision-making regarding the timing of prophylactic surgery based on individual risk stratification. These recommendations emphasize the importance of tailoring the timing of thyroidectomy to the specific risk category assigned to each genetic variant, with the goal of preventing disease progression while minimizing unnecessary intervention. We encountered a case of F-HSCR associated with the germline c.1858T>C (p.C620R) *RET* activating variant in exon 10, which is known to confer moderate risk for MTC. Although only a limited number of MTC cases have been reported in the context of Hirschsprung disease (HD), and it remains unclear whether the management should align with that of MEN2A, we initiated surveillance for MTC in this family. No elevation of key markers, including carcinoembryonic antigen (CEA) or calcitonin, was observed, and no cases of MTC were detected across generations. However, a pheochromocytoma (PHEO) was diagnosed in one family member through screening for plasma-free metanephrines (fMNs). We present our findings in this family and provide a review of relevant literature.

## Introduction

Hirschsprung disease (HD; OMIM #142623) is a congenital disorder characterized by the absence of enteric ganglion cells in segments of the intestine, leading to functional obstruction. Familial Hirschsprung disease (F-HSCR) is often associated with variants in the *RET* proto-oncogene. Approximately 50% of patients with F-HSCR carry a variant in the RET gene, which is also the causative gene of multiple endocrine neoplasia type 2 (MEN2; OMIM #171300) [1-4].

Although *RET* mutations are well-documented in both F-HSCR and MEN2, reports describing their co-occurrence remain rare. Certain activating mutations, particularly those affecting cysteine residues in exon 10 (e.g., codons 609, 611, 618, and 620), are implicated in both conditions, reflecting their complex functional consequences [2,5-6]. Among these, the c.1858T>C (p.C620R) variant is notable, with a reported 13% penetrance for HD and a 20-30% risk for developing pheochromocytoma (PHEO; OMIM#171300) in MEN2A patients [7-10]. However, there is a lack of consensus on whether individuals with F-HSCR and *RET* mutations should undergo surveillance for medullary thyroid carcinoma (MTC; OMIM#155240) or PHEO, particularly when MTC is absent.

Current clinical guidelines primarily focus on MTC surveillance in individuals with *RET* mutations, often recommending prophylactic thyroidectomy based on mutation-specific risk [11]. Yet, the applicability of these guidelines in F-HSCR cases remains uncertain, particularly concerning the monitoring of other MEN2-associated tumors such as PHEO. Moreover, even in families without a history of MTC, the possibility of late-onset manifestations like PHEO should not be overlooked.

In this context, we report a family with F-HSCR harboring the *RET* C620R mutation, in which affected members developed PHEO in the absence of MTC. This case highlights the phenotypic variability associated with *RET* mutations and underscores the need to revisit surveillance strategies to address the full spectrum of MEN2 manifestations. We also review relevant literature to explore the clinical implications for patient care and hereditary risk assessment.

## Case presentation

**Figure 1:**
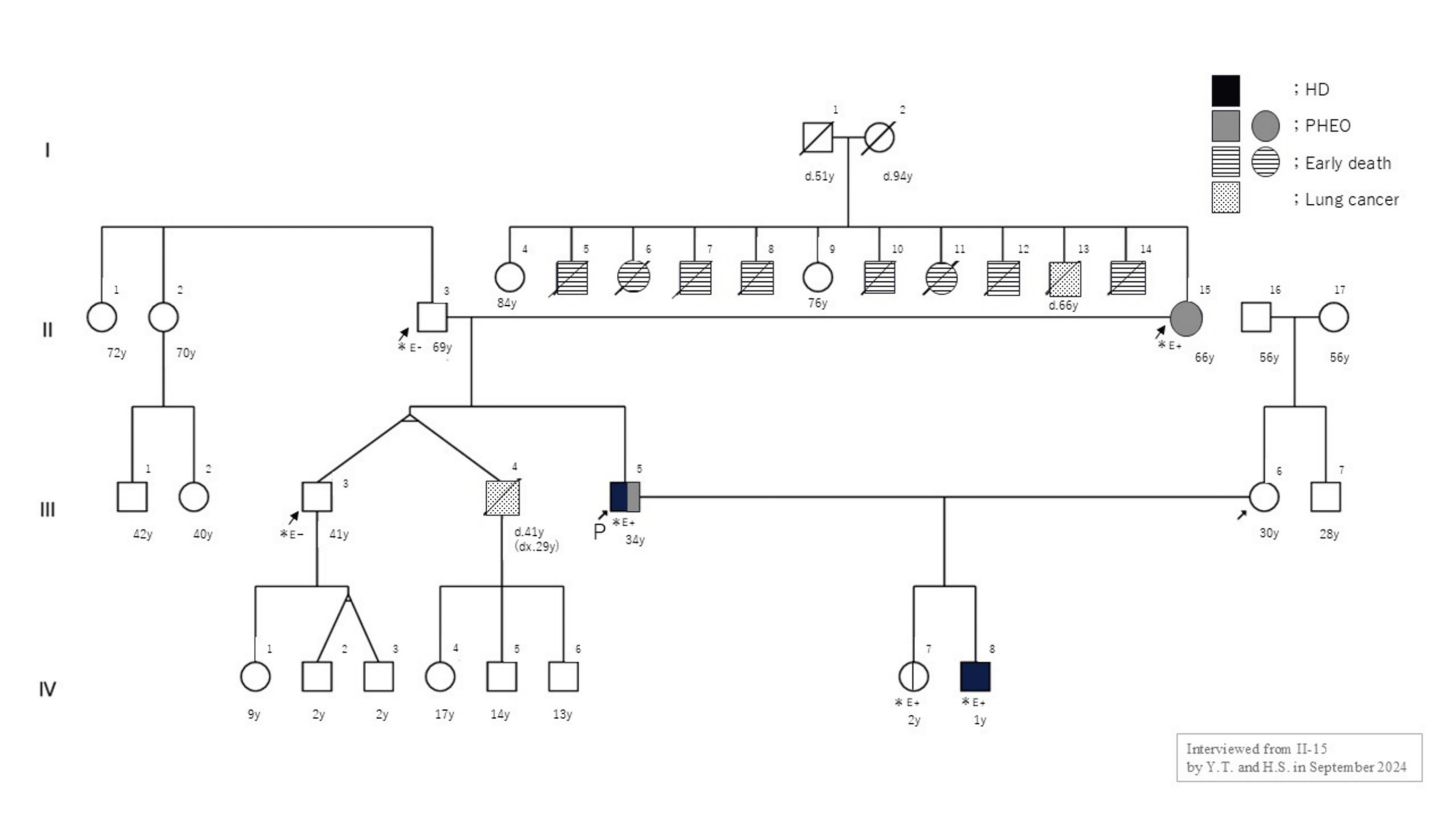
Family tree The figure shows the distribution of genetic mutations in this family. The proband and his son underwent HD, and both were found to share the same pathogenic *RET* variant (NM_020975), c.1858T>C (NP_066124): p.C620R, transmitted from the proband’s mother. The proband’s daughter, who is a carrier of the same variant, has not developed HD. The proband’s mother’s siblings had died in early childhood from unknown causes. HD: Hirschsprung disease; PHEO: pheochromocytoma

A 33-year-old man was diagnosed with total colonic HD at one month of age due to intestinal obstruction and underwent radical surgery (modified Boley’s procedure) at the age of one year. The family pedigree is shown in Figure 1, in which the proband is indicated as individual III-5.

One year after surgery, the patient experienced several episodes of residual intestinal inflammation that resolved with antimicrobial therapy. The patient’s condition was good thereafter, and the examinations were terminated 14 years after the surgery.

His family history was as follows: the maternal uncle died in childhood (details unknown), his other maternal uncle died of lung cancer (details unknown), his older identical twin, who had never been diagnosed with the disease, was diagnosed with lung cancer at 29 years of age and passed away 41 years of age due to brain metastasis.

The proband’s second child, a son, was referred to our hospital for close examination and treatment because he had fed poorly and had experienced repeated episodes of vomiting since birth. We suspected F-HSCR, although it was not confirmed histopathologically. Genetic testing of the proband and his son was performed after genetic counseling during treatment planning. A pathogenic variant of *RET* (NM_020975), c. 1858T>C (NP_066124): p. C620R, was detected in both patients (Table 1).

**Table 1:** Genetic analysis results including ACMG/AMP classification Variant details: This missense variant is located in exon 10 of the *RET* gene, resulting in the substitution of cysteine with arginine at codon 620. Clinical significance: According to ClinVar, this variant is classified as pathogenic and is associated with multiple endocrine neoplasia type 2A (MEN2A). dbSNP ID: The variant is registered as rs77316810 in the dbSNP database. COSMIC ID: In the COSMIC database, it is listed as COSM29084. ClinGen ID: ClinGen identifies this variant as CA008055. Frequency: In the gnomAD database, this variant was not detected among 248,804 control chromosomes, indicating a frequency of 0.000%. ACMG/AMP classification: This variant is classified as pathogenic based on the following criteria: PS1 (strong): Same amino acid change as a previously established pathogenic variant. PM1 (moderate): Located in a mutational hot spot and/or critical and well-established functional domain. PM2 (moderate): Absent from controls (or at extremely low frequency if recessive) in population databases. PP3 (supporting): Multiple lines of computational evidence support a deleterious effect on the gene or gene product. PP5 (supporting): A reputable source recently reports a variant as pathogenic, but the evidence is not available to the laboratory to perform an independent evaluation. ACMG: American College of Medical Genetics and Genomics; AMP: Association for Molecular Pathology

Gene (Reference Sequence)	cDNA Change	Protein Change	Variant Type	Zygosity	Affected Exon	Clinical Significance	dbSNP ID	COSMIC ID	ClinGen ID	Frequency (gnomAD)	ACMG/AMP Classification (Applied ACMG/AMP Criteria)	Associated Disease
RET (NM_020975.6)	c.1858T>C	p.Cys620Arg	Missense	Heterozygous	Exon 10	Pathogenic (ClinVar)	rs77316810	COSM2908	CA008055	0.000% (0/248,804)	Pathogenic (PS1, PM1, PM2, PP3, PP5)	Multiple Endocrine Neoplasia Type 2A (MEN2A)

**Figure 2:**
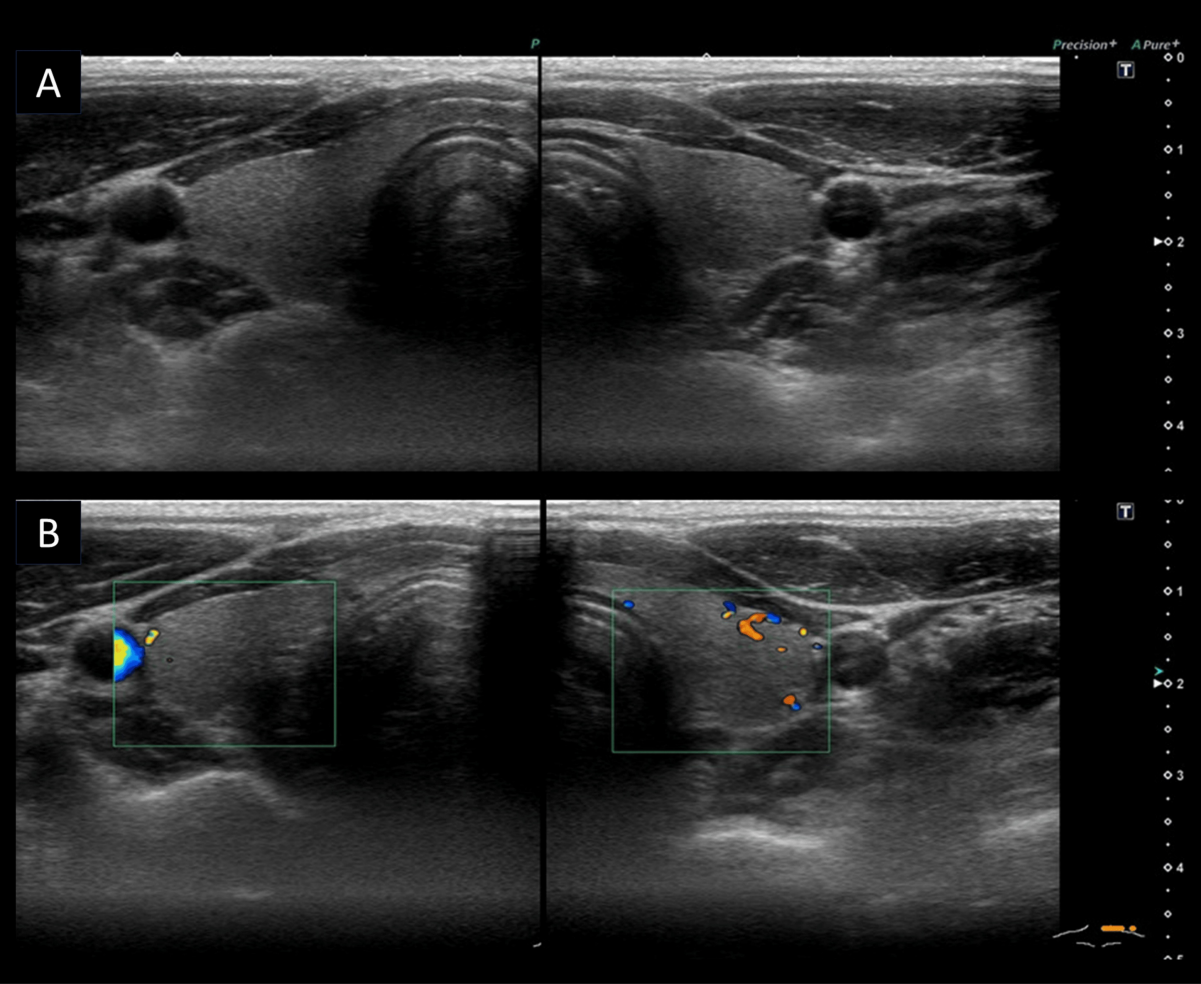
Ultrasound findings of the proband (A) B-mode and (B) color Doppler images showing no abnormal findings in either lobe of the thyroid.

Genetic counseling was conducted for a second time to evaluate the results of the genetic testing. This variant was a moderate risk factor for the development of MTC according to the American Thyroid Association (ATA) risk classification (Table 2), and MTC screening was proposed. A cervical ultrasound examination of the proband revealed no obvious abnormal findings, an intact thyroid parenchyma, no mass lesions, or findings suspicious for C-cell hyperplasia in the upper to middle portion of both thyroid lobes (Figure 2).

**Table 2:** MTC risk classification for RET variants related to HD, only moderate risk extracted (modified from ATA‘s classification) PHEO: pheochromocytoma; HPT: hyperparathyroidism; CLA: cutaneous lichen amyloidosis; MTC: medullary thyroid carcinoma; HD: Hirschsprung disease; ATA: American Thyroid Association

*RET* Mutation	EXON	MTC Risk Level	PHEO	HPT	CLA
C609F/G/R/S/Y	10	Moderate	-10%/20-30%	-10%	N
C611F/G/S/Y/W	10	Moderate	-10%/20-30%	-10%	N
C618F/R/S	10	Moderate	-10%/20-30%	-10%	N
C620F/R/S	10	Moderate	-10%/20-30%	-10%	N

Biochemical testing showed no elevation in carcinoembryonic antigen (CEA), serum calcitonin (3.17 ng/mL and 6.96 pg/mL, respectively), or plasma-free metanephrines (fMNs and plasma-free normetanephrine (fNMN)) (Table 3). Cervical ultrasound and measurement of CEA and calcitonin levels were continued every six months for the next few years.

**Table 3:** Biochemical testing of the proband All test results were within normal limits, showing no abnormalities.

Examination Items	Value	Reference Range	
Carcinoembryonic antigen (CEA)	3.17 (ng/mL)	-5 (ng/mL)	
Calcitonin (Ctn)	6.96 (pg/mL)	Male	Female
		-9.52 (pg/mL)	-6.40 (pg/mL)
Intact parathyroid hormone (iPTH)	34.2 (pg/mL)	18.5-88.0 (pg/mL)	
Plasma-free metanephrine (fMN)	263 (pg/mL)	-130 (pg/mL)	
Plasma-free normetanephrine (fNMN)	166 (pg/mL)	-506 (pg/mL)	

The proband’s youngest son (age 0 years), who had frequent bilious vomiting, abdominal distention, and poor feeding at one day of age, also had the same *RET* variant. A laparoscopic Duhamel operation was performed as radical surgery at eight months of age. A histopathological examination of the colon revealed intestinal aganglionosis, consistent with HD and the long-segment type.

Genetic testing was performed for all relatives who requested familial screening (II-3, II-15, III-4, and IV-7 in Figure 1). Notably, genetic testing of the proband’s parents, who had no significant medical history, was particularly important. If the proband was not an isolated case but had inherited the *RET* mutation, this would serve as a key indicator for assessing the future risk of developing MTC or PHEO in the proband and their offspring, as well as for estimating the age at which they might remain disease-free. The proband’s daughter (IV-7 in Figure 1) and mother (II-15 in Figure 1) carried the same gene variant, whereas the father (II-3 in Figure 1) and elder brother (III-3 in Figure 1) showed no evidence of it (Table 4).

**Table 4:** Pedigree analysis and RET C620R variant status

Family Number (From Figure 1)	Relationship	Age	Sex	*RET* C620R Variant
III-5	Proband	33	M	Positive (Heterozygous)
IV-8	Son	1	M	Positive (Heterozygous)
II-3	Father	69	M	Negative
II-15	Mother	66	F	Positive (Heterozygous)
III-3	Elder brother	41	M	Negative
IV-7	Daughter	2	F	Positive (Heterozygous)

A reassessment of the family history with the proband’s mother revealed that multiple of her siblings had died in early childhood from unknown causes (II in Figure 1).

The mother and the daughter exhibited no thyroid abnormalities, and no elevations of CEA or calcitonin were observed. However, fMN elevation was observed in the mother (192 pg/mL).

**Figure 3:**
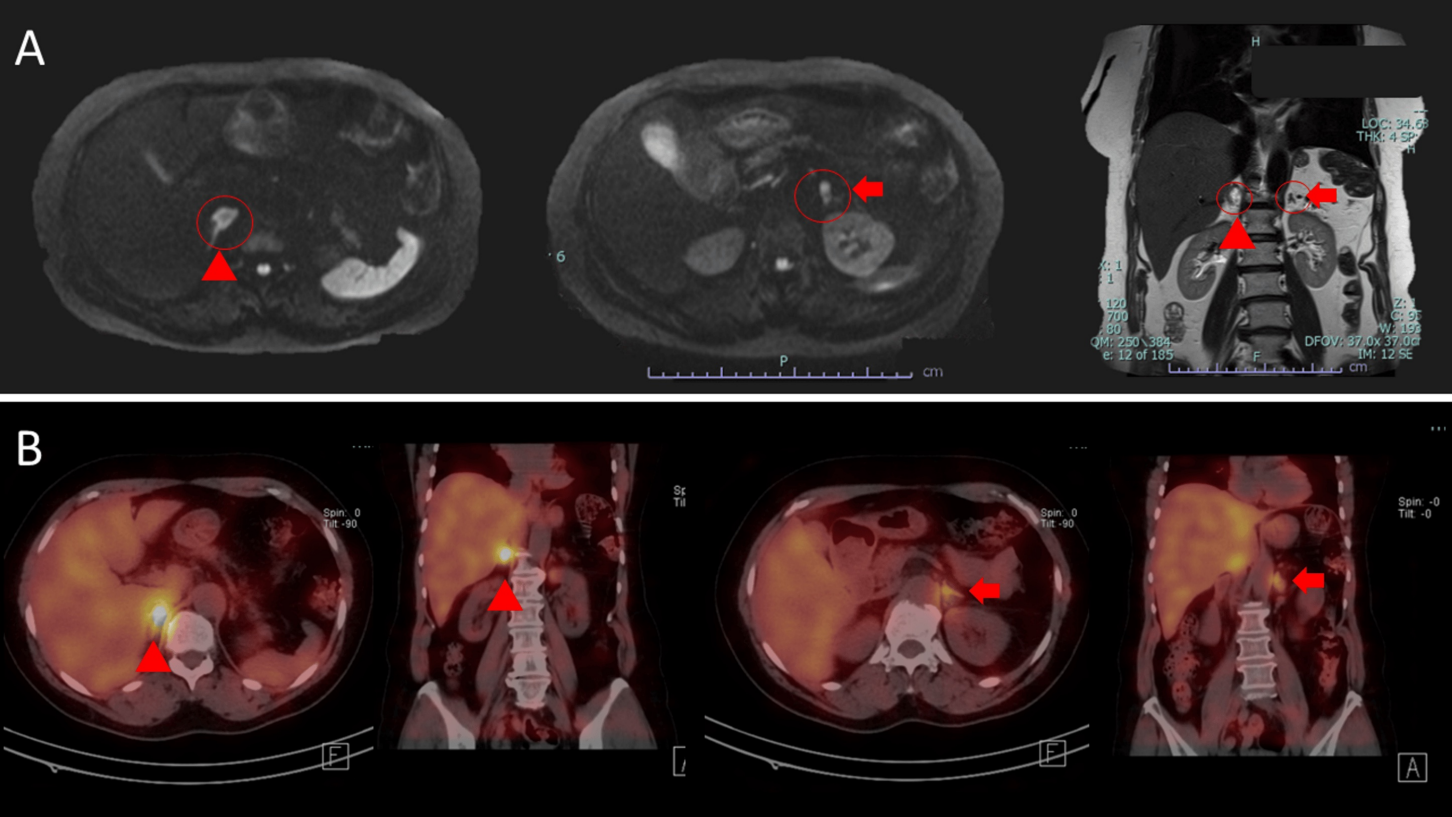
Imaging findings of the proband’s mother (A) Abdominal MRI findings showing an oval mass in front of the right kidney (▲), and a small nodule is also observed on the medial side of the upper pole of the left kidney (➡). (B) ¹³¹I-MIBG scintigraphy findings showing marked uptake in the right adrenal gland (▲), and slight uptake is also observed on the left side (➡), suggesting the possible presence of a lesion. ¹³¹I-MIBG: iodine-131 metaiodobenzylguanidine

Based on these findings, an abdominal magnetic resonance imaging (MRI) was performed, which revealed a mass lesion in the right adrenal gland suspicious for PHEO (Figure 3A). Additionally, the levels of all three catecholamine fractions were elevated (adrenaline, 118 ng/mL; noradrenaline, 521 ng/mL; dopamine, 21 ng/mL) (Table 5). 131I-metaiodobenzylguanidine (131I-MIBG) scintigraphy revealed marked uptake in the right adrenal gland, supporting the diagnosis of PHEO (Figure 3B). During the course of the disease, mild hypertension was observed and managed using oral medication.

**Table 5:** Biochemical testing of the proband’s mother Revealing an elevation in free metanephrine (fMN), with all three catecholamine fractions showing increased levels.

Examination Items		Value (pg/mL)	Reference Range (pg/mL)
Catecholamines, 3 fractionation (plasma)	Adrenaline	118	-100
	Noradrenaline	521	100-450
	Dopamine	21	-20
Plasma-free metanephrines	Free metanephrine	263	-130
	Free normetanephrine	166	-506

**Figure 4:**
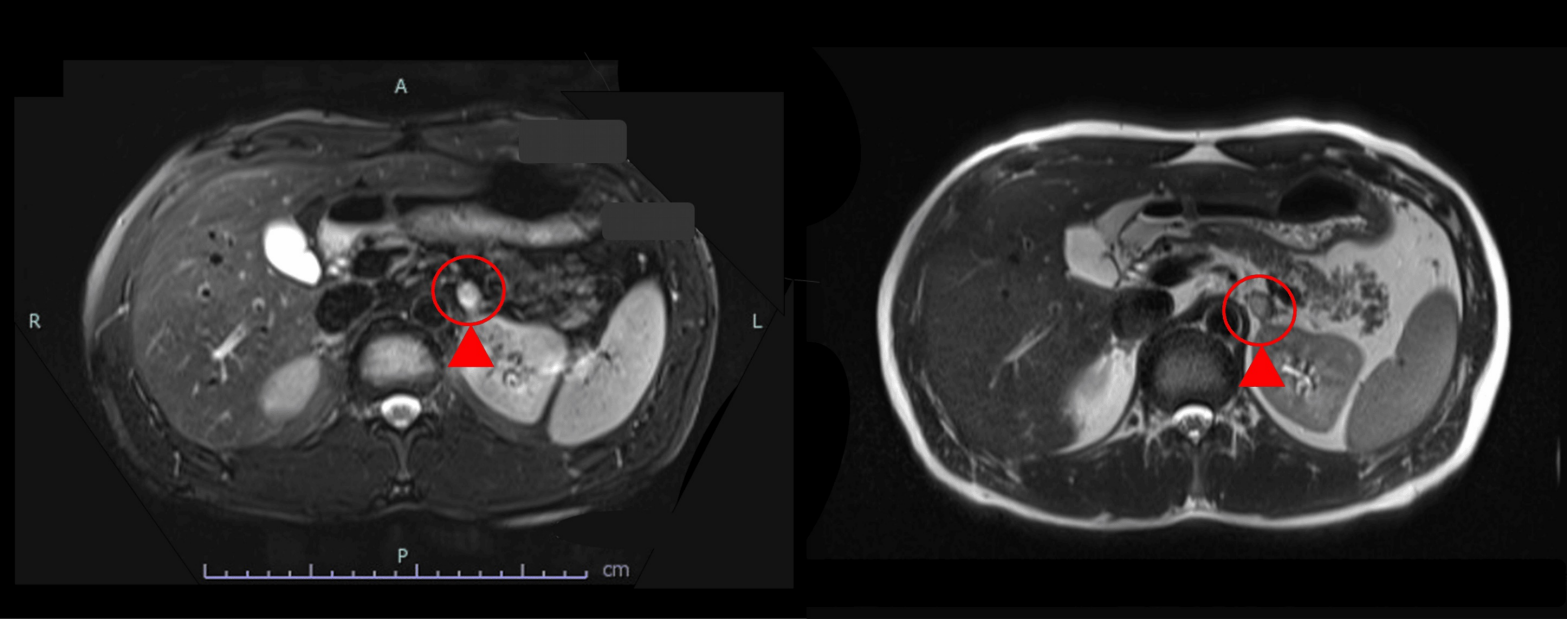
Abdominal MRI findings The findings show an oval mass in front of the left kidney (▲).

To reassess PHEO screening in the proband, abdominal MRI was performed, revealing a 14-mm nodule in the left adrenal gland (Figure 4). The imaging findings were not suggestive of adenoma, prompting further evaluation by adrenal scintigraphy.

We are continuing genetic counseling for this family, including consideration of genetic testing of other relatives (IV-4-6 in Figure 1). Specifically, one of the proband’s twin brothers (III-3 in Figure 1) underwent genetic testing and was found to be negative for the *RET* mutation. Consequently, his children (IV-1-3 in Figure 1) were not considered candidates for genetic testing at this time. The other twin brother (III-4 in Figure 1) had previously passed away due to lung cancer; although they were reported to be monozygotic twins, this has not been conclusively confirmed. If they were dizygotic, there remains a possibility that the deceased brother also carried the *RET* mutation. Therefore, we are considering genetic testing for his three children (IV-4-6 in Figure 1). Notably, the eldest daughter (IV-4 in Figure 1) was hospitalized at the age of four for idiopathic intussusception, which was successfully treated with high-pressure enema reduction, and currently experiences chronic constipation. These gastrointestinal symptoms may suggest a mild form of HD, potentially linked to the *RET* mutation. Given these considerations, we are proceeding with careful evaluation and counseling for this branch of the family.

## Discussion

HD is a congenital disorder in which some intestinal ganglion cells are completely absent and is the most common cause of intestinal obstruction in newborns and infants. It is characterized by high heritability (>80%), the incidence of short-segment disease (80% of HD) is four times greater in males than in females, and there is no sex difference with long-segment HD [4].

It has also been associated with monogenic and chromosomal syndromes. As a single-gene disease, it can be either symptomatic or asymptomatic, with other abnormalities and a variety of inherited forms. *RET* is considered a candidate gene for F-HSCR because a combination of MEN2A and HD has been reported in several families and is expressed in neural crest-derived cells [1-6].

Furthermore, not all individuals carrying the *RET* C620R mutation in our reported family developed clinical manifestations such as HD or PHEO, suggesting incomplete penetrance of this variant. This observation aligns with previous studies indicating that mutations at codon 620 are associated with variable penetrance and expressivity. For instance, while codon 634 mutations (e.g., C634R) are associated with a high penetrance of MTC, often exceeding 90%, codon 620 mutations demonstrate a lower and more variable penetrance for MTC and other associated conditions [5-12]. This variability underscores the importance of individualized risk assessment and further research to elucidate the genotype-phenotype correlations of *RET* mutations.

*RET*, the causative gene of MEN2, was first reported as a proto-oncogene by Takahashi et al. (1985) [2,13]. In 1994, *RET* variants were reported to be associated with HD [3]. In particular, HD is associated with MEN2 and FMTC in families with variants in cysteine codons 609, 611, 618, and 620. These variants are present in approximately 10% of MEN2A families and more than 50% of FMTC families [6,12]. In MEN2A, activating mutations commonly occur at six highly conserved cysteine residues located in exon 10 (codons 609, 611, 618, and 620) and exon 11 (codons 630 and 634), leading to constitutive dimerization and transformation in vitro. Among these, the C620R mutation is notable for its association with both MEN2A and HD, suggesting a dual role in gain- and loss-of-function effects. In contrast, mutations such as C618S are more strongly associated with a higher risk of MTC and less frequently linked to HD. Furthermore, non-syndromic HD is often associated with loss-of-function mutations in *RET*, including missense, nonsense, and frameshift mutations that impair *RET* signaling, leading to aganglionosis in the distal colon without predisposing individuals to MTC or PHEO. Haploinsufficiency is the most likely mechanism, but it is not sufficient to explain its coexistence [2,5,6,9-10]. Even in comprehensive mutation screening for HD, *RET* mutations are compatible with the linkage at the *RET* locus in most families. Specifically, a maximum two-point LOD score of 3.37 (θ̂ = 0.045) was observed between HD and D10S176, a microsatellite marker located on chromosome 10q11.2, indicating significant linkage in a subset of five HD families. However, *RET* mutations have been identified in only approximately 50% of familial cases and 15-20% of sporadic cases [5,6]. Furthermore, linkage disequilibrium (LD) analysis has revealed strong associations between specific single-nucleotide polymorphisms (SNPs) within the *RET* gene and HD. For instance, a haplotype tagging SNP located in intron 1 of the *RET* gene showed a strong association with HD (odds ratio 3.64, 95% confidence interval 2.24-5.92, P < 0.0001) [14]. While most MEN2A patients have been reported to have no intestinal atresia, 2.5-5% of HD patients have *RET* variants, which could have a significant impact on the care of relatives [6]. In the case of MEN2B, more than 90% of the patients were reported to have the M918T variant, and the reported rates of diffuse gangliocytoma (40%) and breast cancer (40%) were also high. Although 40% of MEN2B patients with more than 90% of M918T variants have diffuse gangliocytoma and gastrointestinal symptoms in infancy, only one case of MTC has been reported to be associated with HD [6,14,15]. In MEN2B, the biological significance of missense variants of the *RET* gene is due to loss of function caused by inactivation of the *RET* gene in non-malignant diseases, which accounts for 50% of F-HSCR cases and 10-20% of sporadic cases [4,11]. In contrast, MTC is caused by the activation of the *RET* gene through a gain-of-function, and C620R, which is recognized as a gain-of-function type in the ATA risk classification, has been reported to be inactivated by loss of cell surface expression of the RET protein in vivo [9]. Thus, C620R is considered to have a dual nature: gain-of-function and loss-of-function [6,9,12-14]. It has also been reported that F-HSCR is a multifactorial genetic disease with multiple genetic alterations. The* RET* missense variant identified in HD is minimally pathogenic, and other gene variants may be required for the disease to be expressed [12,15,16].

In cases in which the risk of MTC is considered to be high based on the ATA classification, prophylactic thyroidectomy at a young age is recommended (Table 6); while in cases with a moderate risk of MTC, the malignancy of MTC is lower, MTC does not develop until adulthood, and the option of surgery at approximately five years of age is indicated if the parents wish [2,11,17].

**Table 6:** Management of patients with a RET germline variant detected on genetic screening TTX: total thyroidectomy; Ctn: calcitonin; PHEO: pheochromocytoma; CEA: carcinoembryonic antigen Modified from the American Thyroid Association (ATA) guidelines (Wells et al., 2015) [16].

Risk Level	TTX	Follow-Up After TTX and Surveillance for PHEO
HST (highest)	In the first year or the first months of life	US of the neck, and measurement of Ctn and CEA every six months. Begin screening for PHEO at 11 years of age
H (high)	At or before five years of age, based on serum Ctn levels	
MOD (moderate)	When the serum Ctn level becomes elevated, or in childhood, if the patients do not wish to embark on a lengthy period of evaluation, which might last for years or decades	Follow with evaluation every six months for a year, then annually if Ctn remains undetectable or within normal range. Begin screening for PHEO at 16 years of age.

In a recent report by Wehrli et al., the rate of HD combined with MEN2A was 0.00002%, whereas the rate of HD combined with MTC was 0.000009%. Among 66 MEN2A patients, one (1.5%) had HD. Of the 319 HD patients, one (0.3%) was diagnosed with MEN2A, and of the 839 HD patients, one (0.1%) had MTC, indicating a very low prevalence [18]. The significance of genetic testing for the *RET* (a known cause of MEN2A and MTC) in HD patients was assumed to be prophylactic resection in cases with high to moderate risk variants. This report suggests that the surveillance of relatives with *RET* gene variants in cases of MTC and the approach to suspected MTC/MEN2A in *RET* gene carriers with F-HSCR may need to be reconsidered. Voss et al. reported that it might be desirable to consider the classification by disease onset (early vs. late) rather than by risk (high vs. moderate) because the clinical courses of MTC are similarly aggressive once MTC develops [19]. In contrast, Innella et al. reported that wild-type *RET* patients and carriers of moderate-risk variants differ only by family history, but not by clinical characteristics such as mean age at the time of diagnosis of MTC. The aggressiveness of MTC did not depend on the presence, absence, or type of *RET* variant. The *RET* variant mainly affects the age at onset of MTC, strongly predicts survival, and determines the appropriateness of non-invasive screening recommended for healthy carriers [2].

HD is often diagnosed in childhood, and when an *RET* variant is confirmed by genetic testing, prophylactic resection should be performed cautiously.

We typically assess the presence or absence of *RET* variants to determine the treatment strategy for MTC, a practice now supported by insurance reimbursement. Recently, genetic testing for HD has led to the identification of the *RET* gene, raising the possibility of cases previously not associated with MTC. This suggests that even with the same genetic mutation, such as in MTC families and HD cases, it may be premature to consider similar interventions (e.g., total thyroidectomy in childhood). Moreover, as seen in this case, PHEO could be involved, which might help prevent the exacerbation of unexplained hypertension. This case highlights the utility of genetic testing in families with a suggestive history. Although the *RET *variants identified in both MEN2A-related genetic testing and HD-related testing may be the same, the statistical frequencies of their respective occurrences could differ significantly, depending on the context of the condition.

It is hoped that the accumulation of further data will clarify the appropriate management approach for HD patients with *RET* variants.

## Conclusions

This study underscores the rare co-occurrence of the *RET* C620R mutation in a family with F-HSCR who developed PHEO without MTC. While these findings contribute to the understanding of the genotype-phenotype correlation in *RET* mutations, the conclusions should acknowledge the limitations of a single-family study. The extrapolation of these results to broader populations requires validation through larger, multi-generational studies. Given the potential implications for clinical practice, it is essential to consider surveillance strategies for individuals harboring the *RET* C620R mutation. Recommendations may include regular monitoring for PHEO and other related conditions, tailored to the individual's clinical presentation and family history. Such strategies should be developed in collaboration with genetic specialists and other healthcare professionals. In summary, while the current study provides valuable insights, further research with larger sample sizes is necessary to confirm these findings and to establish evidence-based clinical guidelines for the management of individuals with the *RET* C620R mutation.
